# New Approaches to the Ecology of *Triatoma sordida* in Peridomestic Environments of an Endemic Area of Minas Gerais, Brazil

**DOI:** 10.3390/pathogens14020178

**Published:** 2025-02-11

**Authors:** Carolina Valença-Barbosa, Isabel Mayer de Andrade, Fellipe Dias Tavares de Simas, Ozorino Caldeira Cruz Neto, Nilvanei Aparecido da Silva, Camila Fortunato Costa, Bruno Oliveira Bolivar Moreira, Paula Finamore-Araujo, Marcus Vinicius Niz Alvarez, André Borges-Veloso, Otacílio da Cruz Moreira, Liléia Diotaiuti, Rita de Cássia Moreira de Souza

**Affiliations:** 1 Grupo Triatomíneos, Instituto René Rachou-Fiocruz Minas Gerais, Belo Horizonte 30190-002, MG, Brazil; carolvalencabarbosa@gmail.com (C.V.-B.); belmayer@yahoo.com.br (I.M.d.A.); fellipe.simas@fiocruz.br (F.D.T.d.S.); nilvaneineves1@gmail.com (N.A.d.S.); camilafortunatocosta28@gmail.com (C.F.C.); brunobolivar100@gmail.com (B.O.B.M.); aborgesveloso@gmail.com (A.B.-V.); lileia.diotaiuti@fiocruz.br (L.D.); 2 Plataforma de Análises Moleculares, Laboratório de Virologia e Parasitologia Molecular, Instituto Oswaldo Cruz/Fiocruz, Rio de Janeiro 21040-360, RJ, Brazil; paulafinamore@gmail.com (P.F.-A.); otacilio@ioc.fiocruz.br (O.d.C.M.); 3 Centro de Endemias e Zoonoses, Secretaria de Saúde, Espinosa 39510-000, MG, Brazil; ozorinoneto@gmail.com; 4 Instituto de Biotecnologia de Botucatu, Universidade Estadual Paulista, São Paulo 18607-440, SP, Brazil; marcus.alvarez@unesp.br

**Keywords:** *Triatoma sordida*, Triatominae, *Trypanosoma cruzi*, Chagas disease, blood meal, discrete typing unit, parasite load, peridomicile, kissing bug

## Abstract

*Triatoma sordida* is a native South American species and the most frequently captured triatomine in artificial environments in Brazil. Although considered a secondary vector of *Trypanosoma cruzi*, it is typically associated with low infection rates. To investigate its role in an endemic area for Chagas disease in northern Minas Gerais, Brazil, we employed a multidimensional approach that combined triatomine capture data with quantitative and qualitative analyses of *T. cruzi*. A total of 1861 *T. sordida* specimens were captured, of which 1455 were examined and 210 (14.4%) were found to be infected with *T. cruzi*. The most prevalent discrete typing unit (DTU) was TcI (80%), followed by TcII (8%), TcV (5%), and TcIII (3%). Molecular techniques provided new insights into the ecology of *T. sordida*, revealing a higher infection rate than previously reported and a parasitic load lower than that observed in other quantified species. Chickens were confirmed as the primary food source, playing an epidemiological role in maintaining infected insects with four *T. cruzi* DTUs. The observed diversity of *T. cruzi* DTUs suggests a lack of environmental segregation, likely due to the extensive movement of various host species between wild and domestic habitats, resulting in overlapping transmission cycles.

## 1. Introduction

Triatomines (Hemiptera: Reduviidae) are blood-sucking insects and vectors of the protozoan *Trypanosoma cruzi*, the causative agent of Chagas disease (ChD). It is a neglected tropical disease (NTD) and one of the most significant infectious diseases in Latin America, affecting approximately 6–7 million people [1]. The disease has significant social and economic impacts, as it remains incurable in its chronic phase. Additionally, its acute phase is predominantly asymptomatic, which makes diagnosis even more challenging. The primary measure to reduce the incidence of new infections is by vector control [1].

Although vector control measures have significantly reduced the risk of *T. cruzi* transmission in Brazil through extensive campaigns aimed at eliminating house infestation by the non-native species *Triatoma infestans*, the risk of parasite transmission by autochthonous vectors persists, as they cannot be eradicated [2,3]. More than 150 triatomine species have been formally described, with 67 distributed across the 27 states of Brazil [4,5,6]. Four species—*Panstrongylus megistus*, *Triatoma brasiliensis*, *Triatoma pseudomaculata*, and *Triatoma sordida*—warrant considerable attention due to their epidemiological importance, as they are frequently found in and around human dwellings [7,8].

*Triatoma sordida* [9] is a native triatomine species in South America, considered synanthropic in Brazil. This taxonomy is under review. Currently, it belongs to the *T. sordida* subcomplex, a monophyletic group, comprising *T. sordida*, *Triatoma rosai*, *Triatoma garciabesi*, *Triatoma jurbergi*, *Triatoma matogrossensis*, and *Triatoma vandae* [10]. Considering the species *T. sordida*, *T. patagonica*, *T. guasayana*, and *T. garciabesi*, cryptic speciation has been suggested [11]. However, some authors argue that the term “cryptic speciation” does not apply, as these species exhibit morphological and/or morphometric differences [12]. It is currently the most commonly captured triatomine in peridomestic Brazilian environments [13,14,15], where it has been reported to be naturally infected with *T. cruzi* [15]. This species occupies several natural ecotopes, primarily found under the bark of live or dead trees [16] and, to a lesser extent, on the trunks of several palm trees species [17]. Around and near houses, *T. sordida* can form numerous colonies, frequently found colonizing chicken coops and feeding on these animals [16,18,19]. Additionally, *T. sordida* has been found living in association with various mammals, such as rodents and opossums [16,20], which are known as potential wild reservoirs of *T. cruzi* [21]. In this regard, understanding the ecology, behaviour, and infection dynamics of this vector is essential for developing effective control strategies.

Artificial ecotopes, such as the annexes in peridomestic areas, play a crucial role in facilitating interactions between the wild and domestic populations of triatomines and *T. cruzi* genotypes [22,23]. Given that this triatomine species inhabits both natural and artificial environments, the interaction between wild and domestic cycles may be possible [24], posing challenges to control efforts. Furthermore, anthropogenic alterations to the landscape, such as uncontrolled grazing and deforestation, contribute to the concentration of wild mammals and triatomines in peridomestic areas, thereby establishing local transmission cycles of *T. cruzi* [25].

The ecological history of the *T. cruzi* strain distribution has been shaped significantly by mammalian migrations in the Americas [26]. The six parasite genotypes known as discrete typing units (DTUs)—TcI to TcVI—exhibit considerable genetic variability, complicating our understanding of transmission cycles [27,28]. Currently, no associations have been established between parasite genotypes and biological response variables, such as the biome, environment, or host species [29]. Also, it is important to highlight that the parasitemia of *T. cruzi* in mammalian hosts is an important factor in the parasitic infection of vectors [29].

Some mammals associated with synanthropic triatomine species, such as *T. sordida*, act as a link between environments and *T. cruzi* cycles [30,31]. The integration of the extra-domestic population of *T. sordida* into the domestic transmission cycle of the parasite has been reported in Minas Gerais, Brazil [16]. This epidemiological scenario, combined with the high number of specimens captured in households, requires significant efforts from the health system to maintain control. To date, there are no reports on the characterization of *T. cruzi* genotypes in *T. sordida*, and few studies have focused on the blood meal source using precipitin tests [16,20]. Although this method has proven useful, it is either inadequately sensitive or specific [32].

In this study, we employed a multi-dimensional approach combining field survey data of triatomines and both quantitative and qualitative analyses, including blood meal source, parasitic load, and *T. cruzi* DTUs infecting *T. sordida* collected in Espinosa, Minas Gerais, Brazil. Our aim was to advance the understanding of the importance of this vector, highlighting the risk factors associated with potential new *T. cruzi* transmission in domestic environments within this region.

## 2. Materials and Methods

### 2.1. Collection of Triatoma sordida

Active searches for triatomine insects were conducted in the municipality of Espinosa (14°55′33″ S; 42°49′08″ O), Minas Gerais, Brazil, during routine entomological surveillance surveys. Five localities were investigated, namely Curral Velho, Sítio do Limoeiro, Barro Vermelho, São Pedro, and Coelheiro. The surveys were carried out between 2018 and 2019 by endemic disease control agents in domiciliary units (DUs), including all annexes in the peridomicile. Peridomestic ecotopes were defined as areas surrounding residences, such as shelters for domestic animals, piles of wood and tiles, and other outbuildings [33]. Triatomine insects were captured manually using tweezers. Collected specimens were examined as described below and individually stored at 20 °C in microtubes containing 70% ethanol until DNA extraction.

After the captures, insecticide spraying was carried out in triatomine-infested domiciliary units (intra- and peridomicile) by endemic disease control agents.

### 2.2. Natural Infection by Trypanosoma cruzi

To detect natural infection by *T. cruzi*, rectal content feces were collected by abdominal compression, placed on a slide with saline solution and covered with a glass coverslip (size 22 mm × 22 mm). Samples were examined at the Instituto René Rachou-Fiocruz Minas using a binocular optical microscope at 400× magnification, screening all fields for *T. cruzi*. Rectal contents testing positive for *T. cruzi* by optical microscope were directly cultured in liver infusion tryptose (LIT) medium supplemented with 10% fetal bovine serum and incubated at 38 °C [34]. Cultures were monitored daily for the first seven days using standard light microscopy. Positive cultures were aliquoted and stored at −20 °C until DNA extraction. Additionally, the natural infection index by *T. cruzi* was calculated (number of infected triatomines × 100/number of insects examined).

For *T. cruzi* molecular diagnosis, DNA samples of *T. sordida* were submitted to kDNA-PCR. The set of primers used in the conventional PCR assays were forward 121 (5′-AAATAATGTACGGG(T/G)GAGATGCATGA-3′) and reverse 122 (5′-GGTTCGATTGGGGTTGGTGAATATA-3′), as designed by Sturm et al. [35] and Wincker et al. [36]. The PCR was performed following conditions previously described [31].

### 2.3. DNA Extraction

The intestine (midgut, hindgut, and rectum) of each *T. sordida* specimen was macerated using a sterile crusher, and genomic DNA was isolated using the DNeasy Qiagen^®^ kit in accordance with the protocol provided by the manufacturer. In addition, DNA was extracted from the positive in vitro cultures. Quantification of the extracted DNA samples was determined using a NanoDrop™ One Microvolume UV-Vis Spectrophotometer (Thermo Scientific, Waltham, MA, USA). All DNA samples were stored at −80 °C until amplification by polymerase chain reaction (PCR).

### 2.4. T. cruzi Genotyping

The molecular characterization of *T. cruzi* DTUs from the DNA of abdominal contents and in vitro culture was performed based on multilocus PCR analysis [37]. For genotype identification, PCRs using three biomarkers (spliced leader, 24 Sα rDNA, and nuclear fragment A10) were conducted. Each biomarker presents a distinct PCR profile, allowing for DTU differentiation. PCR products were electrophoresed on 6% polyacrylamide gels and stained with silver nitrate. The identification of *T. cruzi* DTUs was performed according to the procedures described by the authors [37].

### 2.5. T. cruzi Quantification by Real-Time PCR (qPCR)

Quantification of the parasitic load was performed [38]. The qPCR assays utilized a multiplex Taqman system targeting the *T. cruzi* nuclear satellite DNA (sat-DNA) [39] and the mitochondrial 12S rRNA gene of triatomines [38]. Standard calibration curves for *T. cruzi* and triatomine targets were constructed by serially diluting total DNA obtained from non-infected triatomine intestine samples (adults *T. sordida* from insectary) supplemented with 10^5^ *T. cruzi* epimastigotes (Dm28c clone, TcI). The resulting DNA was serially diluted to a range of 10^5^ to 0.5 *T. cruzi* equivalents and 5 to 0.002 triatomine intestine unit equivalents.

### 2.6. Identification of Feeding Sources

DNA samples from intestines of non-starving triatomines were used to identify feeding sources through direct sequencing of the vertebrate 12S rRNA locus (L1085: 5′-CCCAAACTGGGATTAGATACCC-3′; and H11259: 5′-GTTTGCTGAAGATGGCGGTA-3′) [40]. Amplicons were purified using the Wizard^®^ SV gel and PCR Clean-Up System kit (Promega, Madison, WI, USA), then sequenced in both directions using PCR primers. DNA sequencing reactions employed the BigDye^®^ Terminator v.3.1 Cycle Sequencing Kit (Applied Biosystems, Foster City, CA, USA) and were processed on an ABI 3730 Sequencer. Bi-directional sequences were assembled and edited using SeqMan (DNASTAR software package, version 12.3, DNASTAR Inc., Madison, WI, USA). The consensus sequences generated were compared with previously published sequences using BLASTN hosted on the NCBI server (http://www.ncbi.nlm.nih.gov/BLAST) (accessed on 10 February 2025).

### 2.7. Statistical Analysis

Statistical analysis was performed using R [41] and Prisma software (version 9). Parasite loads between TcI and TcII were compared using the Wilcoxon–Mann–Whitney test. Additionally, the food sources of domestic and wild animals identified in triatomine vectors were analyzed. The Kruskal–Wallis test, followed by Dunn’s post hoc test, was applied to evaluate log-transformed parasite loads across different vertebrate blood meal sources, as well as single and mixed *T. cruzi lineages* identified in triatomine vectors and their evolutionary stages. McNemar’s test was used to compare the two detection methods (morphological and molecular) for *T. cruzi*.

## 3. Results

### 3.1. T. sordida Captures and T. cruzi Infection Rates

Triatomines were found in 140 (48%) out of 294 domiciles investigated. A total of 1861 specimens were captured across the five localities, with 84 (5%) found indoors and 1777 (95%) in peridomestic environments. Specifically, 1485 (84%) were captured in chicken coops, 114 (6%) in pigsties, 88 (5%) in wood piles, 61 (3%) in tile piles, 14 (0.8%) in bricks and tiles, nine (0.5%) in brick piles, and six (0.3%) in corrals. Colonies of *T. sordida* of varying sizes were recorded, some exceeding 400 specimens. Although stage V nymphs and males predominated, triatomines of all developmental stages were captured, including indoors (Table 1).

Overall, 1455 *T. sordida* were examined by an optical microscope and/or kDNA-PCR, with 210 (14.4%) found to be infected by *T. cruzi* (Table 2). Using only an optical microscope, 1357 fecal samples were examined, with 56 (4%) testing positive for *T. cruzi*. Using the kDNA-PCR technique alone, 985 samples were analyzed, with 201 (20%) showing the expected bands for *T. cruzi*. A comparison of 887 samples analyzed by both an optical microscope and kDNA-PCR revealed that 47 (5%) and 168 (19%) were positive for *T. cruzi*, respectively. McNemar’s test indicated a significant difference between the results of these two methods (χ^2^ McNemar = 119, *p*-value < 0.0001).

Considering the capture environment and ecotopes, the largest infection index was observed in triatomines collected in piles of bricks, followed by those found in pigsties, chicken coops, wood piles, tile piles, and indoors (Table 2).

### 3.2. T. cruzi Genotyping and Detection of Mixed Infections

Out of 201 kDNA-PCR-positive samples of *T. sordida*, we identified the parasite genotype in 143 (71%) samples. The most prevalent DTU was TcI (80%), followed by TcII (8%), TcV (5%), and TcIII (3%). Additionally, we detected mixed infections of TcI and TcII and TcIII and TcV in five (4%) bugs (Table 3). Regarding the DTUs and the capture ecotopes, all DTUs were found in pigsties and chicken coops, which can be attributed to the greater number of samples analyzed from these ecotopes (Table 3).

### 3.3. T. cruzi Parasite Load in T. sordida by Real-Time PCR (qPCR)

**Figure 1:**
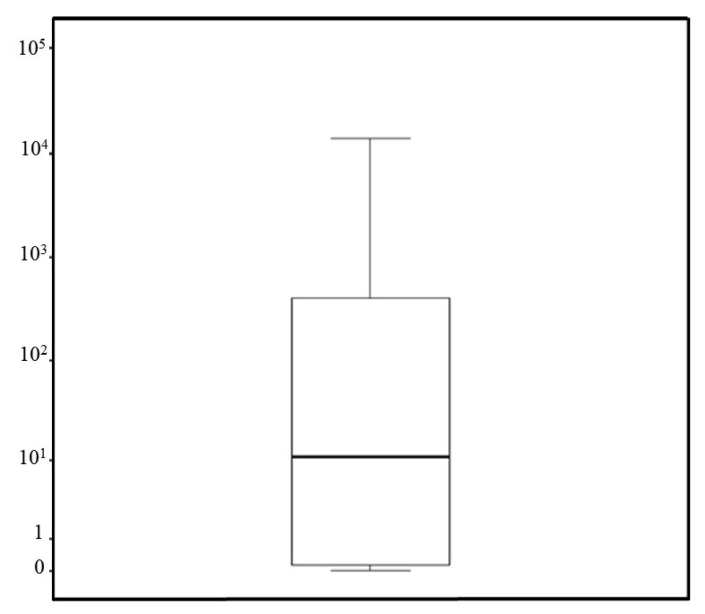
Boxplots of *T. cruzi* parasite load in *T. sordida* captured in the rural region of Espinosa, MG, Brazil.

The quantification of the *T. cruzi* parasite load was performed on 143 *T. sordida* specimens infected with *T. cruzi*, achieving successful quantification in 68 specimens. Of these, one (1%) was in the N2 stage, seven (10%) in N3, three (4%) in N4, 39 (57%) in N5, and 11 (16%) were males and seven (10%) were females. The dynamic range for the *T. cruzi* load quantification in *T. sordida* intestine samples extends from 10^4^ to one parasite equivalent, and from 0.0001 to one intestine equivalent. This dynamic range provided linear quantification over a four-log range for *T. cruzi* and six-log range for triatomines, allowing for an accurate standardization of parasite loads. The linearity coefficients (R^2^) were 0.99 for both targets. PCR efficiencies were 86.5% for the *T. cruzi* nuclear satellite DNA target and 83.2% for the triatomine 12S ribosomal subunit target, confirming the enhanced performance of the assay. The observed median value for the parasitic load was 10 *T. cruzi*/intestine equivalent (Figure 1).

### 3.4. Blood Meal Identification in T. sordida Specimens

Out of 187 DNA samples tested for blood meal identification, 175 (94%) sequences were obtained. The majority of these were from N5 nymphal instars (62%), followed by adults (18%), N4 (8%), N3 (9%), N2 (0.6%), and N1 (0.6). Additionally, 12 sequences exhibited double peaks in the electropherograms, indicating mixed blood meals. The sequences generated from 174 *T. sordida* showed high identity (98–100%) to sequences of *Gallus gallus* (N = 141, 80%), *Rattus rattus* (N = 14, 8%), *Thrichomys apereoides* (N = 7, 4%), *Sus scrofa* (N = 6, 3%)*, Wiedomys cerradensis* (N = 2, 1%)*, Canis lupus familiaris* (N = 2, 1%)*, Capra hircus* (N = 1, 0.6%), and *Felis catus* (N = 1, 0.6%), available in the NCBI database. However, one sequence returned to a BLAST identity of 97% with a sequence in Genbank (MN206976.1). It is likely that this insect fed on the mustelid *Galictis cuja*, since there are no 12S rRNA sequences for this mammal in the database.

**Figure 2:**
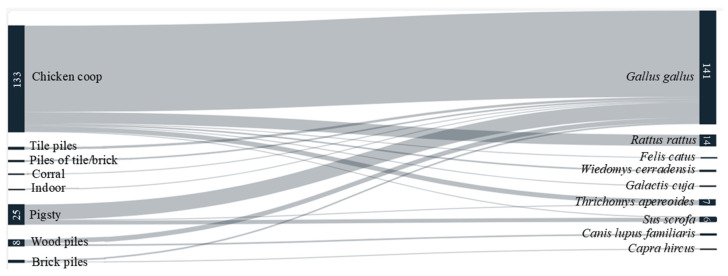
Sankey diagram illustrating the relationships between ecotopes and identified blood meal sources in *T. sordida* collected from rural areas of Espinosa, MG, Brazil.

We also assessed the food source for *T. sordida* in relation to the ecotopes where the specimens were captured. As expected, most triatomines that fed on *G. gallus* were collected in chicken coops, although other food sources were also detected in this ecotope. Notably, all insects fed on *R. rattus* were captured in chicken coops, as with other rodents and mammals such as *F. catus*, *W. cerradensis*, *G. cuja*, *T. apereoides*, and *S. scrofa*. Specimens captured indoors had fed primarily on *G. gallus* (Figure 2).

### 3.5. Occurrence of DTUs in T. sordida After Feeding on Different Hosts

The most common blood meal source in the subsample with determined DTUs was *G. gallus*, accounting for 80%, followed by *R. rattus* (8.9%), *T. apereoides* and *S. scrofa* (3.3% each), *C. lupus familiaris* (2.2%), and *F. catus* and *C. hircus* (1.1% each) (Table 4). For the three *T. sordida* that fed on *W. cerradensis* (N = 2) and *G. cuja* (N = 1), the infecting DTU could not be identified.

### 3.6. Correlation of DTUs and the Parasite Load in T. sordida

**Figure 3:**
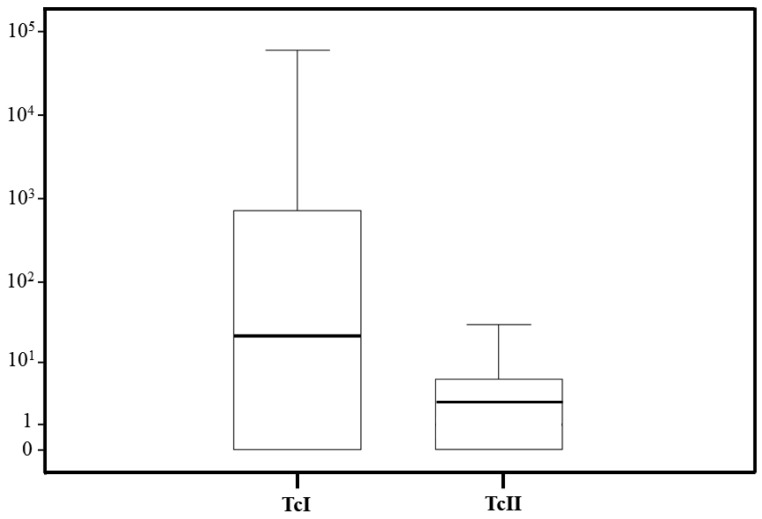
Boxplot comparing estimates of the *T. cruzi* parasite load according to the infecting *T. cruzi* DTU lineage in *T. sordida* from the rural region of Espinosa, MG, Brazil.

Quantification assays using qPCR revealed parasite loads in 47 *T. sordida* infected with TcI, nine infected with TcII, two infected with TcIII, one infected with TcV, and three infected with mixed DTUs. Due to the small sample sizes, comparisons were only made between TcI and TcII. The highest observed parasite loads were 6.1 × 10^4^, 1.2 × 10^2^, and 48 for TcI, TcII, and mixed infections, respectively. The lowest parasite loads were 0.0001, 0.0004, and 0.008, respectively. Median parasite loads were 22 for TcI, 4.8 for TcII, and 5.8 *T. cruzi*/intestine equivalents for mixed infections. No significant differences were observed in *T. cruzi* load between TcI and TcII (*p*-value = 0.506) or for mixed infections (*p*-value = 0.315) (Figure 3).

### 3.7. T. cruzi Parasite Load According to the Food Source

**Figure 4:**
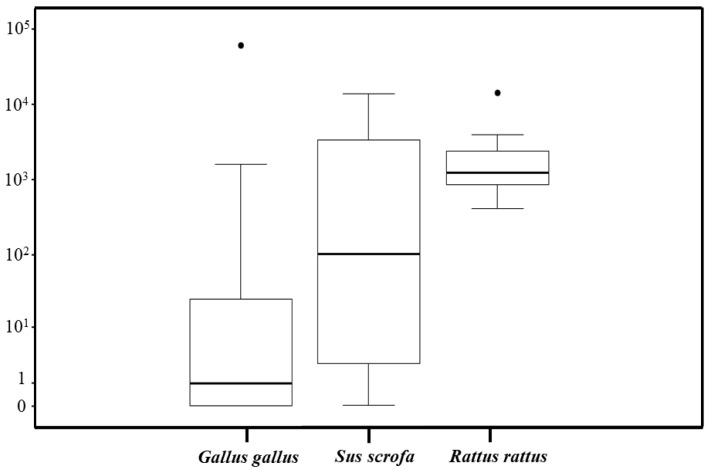
Boxplot comparing estimates of the *T. cruzi* parasite load according to the blood meal sources identified in *T. sordida* from the rural region of Espinosa, MG, Brazil.

A total of 43 triatomines that fed on *G. gallus*, seven on *R. rattus,* four on *Sus scrofa*, two on *C. lupus familiaris*, and one on *T. apereoides* were successfully quantified for parasite load. For statistical analyses, we compared *T. cruzi* parasite loads between *T. sordida* fed on *G. gallus*, *R. rattus*, and *S. scrofa* due to the sample size limitations. Insects fed on *R. rattus* exhibited significantly higher *T. cruzi* loads compared to those fed on *G. gallus* (*p* = 0.0007) (Figure 4). No significant differences were observed in comparisons between *G. gallus* and *S. scrofa* or *S. scrofa* and *R. rattus*. The median *T. cruzi* loads were 0.6, 1.2 × 10^3^, and 4 × 10^2^ parasites/intestine equivalents for *T. sordida* fed on *G. gallus*, *R. rattus*, and *S. scrofa*, respectively (Figure 4).

### 3.8. T. cruzi Parasite Load According to Triatomine Stage

**Figure 5:**
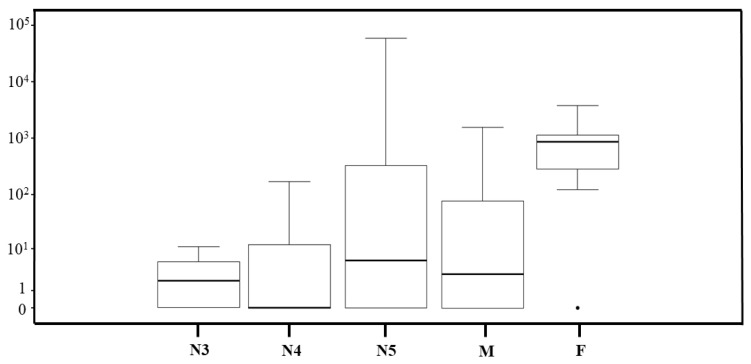
Boxplot comparing the estimate of the *T. cruzi* parasite load according to the development stage of *T. sordida* from the rural region of Espinosa, MG, Brazil.

*T. sordida* females had significantly higher *T. cruzi* loads compared to N3 (*p*-value = 0.012), N4 (*p*-value = 0.018), and N5 (*p*-value = 0.024) nymphal instars and males (*p*-value = 0.014). The median values for parasitic loads were 1.8, 0.008, 5.8, 2.8, and 8.9 × 10^2^ *T. cruzi* per intestine equivalents for N3, N4, N5, males, and females, respectively (Figure 5).

## 4. Discussion

Our study provides novel insights into the infections of *T. sordida* by *T. cruzi* in an endemic area of Minas Gerais, Brazil, underscoring the significance of this vector in the peridomestic transmission cycle of the parasite. The principal findings of this study are as follows: (i) a high natural infection rate of *T. cruzi* in *T. sordida*; (ii) a relatively low quantified parasitic load of *T. cruzi*; (iii) specimens that fed on *R. rattus* exhibited the highest *T. cruzi* load per intestine, whereas those that fed on *G. gallus* showed the lowest; (iv) a notable diversity of *T. cruzi* DTU and identified blood meals; (v) no significant difference in *T. cruzi* load between insects infected with TcI and TcII; (vi) females exhibited the highest parasitic load compared to other development stages.

Although *T. sordida* is the most frequently captured triatomine species in Brazil, including in the northern Minas Gerais state, few studies have focused on its natural *T. cruzi* infection rate [13,16,42]. Moreover, due to the low *T. cruzi* infection rate, this vector has been considered to have a low potential for sustaining high rates of *T. cruzi* transmission [42,43,44]. However, our findings present a different scenario, particularly when considering results obtained using molecular methods. The significant difference between *T. cruzi* detection techniques (optical microscope and kDNA-PCR) was anticipated, as extensively reported in the literature, with parasitic load being a critical factor for accurate diagnosis [39,45,46]. According to Valença-Barbosa et al. [31], triatomine intestinal samples with 10^3^ *T. cruzi* per intestine have a 30% chance of being detected as positive by an optical microscope. Considering that the *T. sordida* population examined in our study exhibited a low parasitic load, with a median value of 10 parasites/intestine, false negative results by optical microscope are expected. It is well known that this method tends to underestimate detection rates [39,45,46]. Additionally, it is important to note previous studies evaluating *T. cruzi* infection in this species employed the optical microscope technique, which may explain the lower infection rates reported compared to our findings.

To date, no study has evaluated or defined “high” or “low” parasitic load of *T. cruzi* in different triatomine species and their implications for vector transmission. A few studies have reported a wide range of parasitic load values from 3.02 × 10^5^ to 1.29 × 10^9^, 0.13 to 2.2 × 10^10^, and 3.94 to 7.66 × 10^6^ *T. cruzi* per intestine for *Mepraia spinolai* (Chile) [47], *Triatoma melanica* (Brazil) [31], and *Triatoma brasiliensis* (Brazil) [30], respectively. *T. melanica* and *T. sordida* are distributed throughout the northwest of the Minas Gerais state, along the border between Bahia and Minas Gerais, in a transition area between the Caatinga and Cerrado biomes [48]. However, these species occupy different ecotopes. *T. melanica*, which is exclusively sylvatic, is associated with rocky crevices in the Espinhaço mountain range, whereas *T. sordida* in Brazil is arboreal, primarily found under tree bark, and is frequently captured inside and around human dwellings [16].

Our *T. sordida* population exhibited a *T. cruzi* load 10 times lower than that reported for *M. spinolai*. This difference in parasitic load may be attributed to the preferred food sources of these triatomine species. Approximately 84% of *T. sordida* were captured in chicken coops and, consequently, 80% fed on chicken, highlighting their feeding preference for birds, as previously reported [16,49,50]. Chickens are refractory to *T. cruzi* infection, as their serum is resistant to trypomastigotes [51,52]. However, chickens play an important role in the survival and maintenance of triatomine populations already infected by *T. cruzi* [28].

*T. sordida* is considered a ubiquitous species with a broad ecological range, occupying various ecotopes and feeding on diverse food sources, including rodents and other mammals. In this study, the majority of insects (96%) fed on rodents (*R. rattus*, *W. cerradensis*, and *T. apereoides*) even when captured in chicken coops. These mammals often invade peridomestic areas [53] attracted by chicken food, which may help explain the *T. cruzi* infection rate in this triatomine population. Infestations of *T. sordida* in peridomestic structures in Minas Gerais have frequently been associated with chicken coops, as well as corrals and wood piles [16,43,44], as observed in the present study.

We identified seven different mammals as food sources, including wild and synanthropic rodents, domestic and farm animals (cats, dogs, pigs and goats), and another wild mammal, the lessor grison. Previously, our group reported the presence of food content in *T. melanica*, resembling the mustelid *G. cuja* in the same study area [31]. However, no 12S rRNA sequences for this mammal are available in the database. Most triatomine species are capable of feeding on the blood of a wide diversity of vertebrates, both wild and domestic, which contributes to their adaptation to various habitats [54]. Regarding domestic animals, particularly dogs and cats, they are key reservoir hosts of *T. cruzi*, bridging the gap between the domestic and wild transmission cycles of the parasite [55,56,57]. Both cats and dogs are epidemiologically important sources of infection for bugs and householders. The movement of domestic cats into wild environments due to human activities underscores the connection between domestic and wild environments [58]. *T. sordida* uses similar native mammals as food sources, much like *T. melanica*, including *T. apereoides*, *W. cerradensis*, and *G. cuja* [31], suggesting intense movement of these mammals between areas. Also noted was this movement of biological elements, including *T. cruzi* reservoirs, driven by anthropogenic actions, which facilitates the reciprocal (sylvatic-peridomestic) circulation of the parasite [58]. For wild mammals, such as rodents, their epidemiological importance as reservoirs of *T. cruzi* is well known [59]. Additionally, all these food sources were identified in specimens from various peridomestic ecotopes, indicating the diversity and intense circulation of animals, which act as a link nature and human-made environments.

Moreover, some *T. sordida* specimens that fed on *R. rattus* exhibited the highest parasitic loads, emphasizing the significant role of these mammals in the transmission cycles of *T. cruzi*. This rodent has been previously identified as an important domiciliary and peridomiciliary reservoir for the parasite [60,61]. As a synanthropic species, *R. rattus* occupies both natural and artificial habitats, facilitating the introduction of *T. cruzi* into transmission cycles near human dwellings. The parasitemia of mammalian hosts is a crucial factor in vector infection [29]. It is important to note that directly associating parasitic load with parasitemia in animals remains challenging due to various influencing factors [28]. However, the dominance of a particular blood source can influence the dynamics of *T. cruzi* transmission cycles [62]. In our study, bugs that fed on chicken exhibited the lowest parasitic loads, highlighting their ability to feed on multiple hosts. While birds do not participate in *T. cruzi* transmission, once triatomines feed on an infected mammal, the infection can persist for a long time [63]. Furthermore, in this study, humans were not identified as a food source for this triatomine species. A plausible explanation for this absence is that most of the insects analyzed were collected in peridomestic ecotopes, where an abundant animal food source is available, while humans are only transient in this environment. Moreover, *T. sordida* is considered a predominantly peridomestic species [18].

This report provides a comprehensive characterization of *T. cruzi* genotypes in *T. sordida* from Brazil. We observed a great diversity of DTU in this triatomine population. However, the parasitic load was not significantly affected by genotypes. TcI was the most prevalent DTU, followed by TcII, TcV, and TcIII.

TcI is the most prevalent genotype in the Americas, predominantly associated with hosts from the orders Didelphimorphia and Rodentia [64]. Moreover, TcI has already been reported infecting *T. sordida* individuals that invade houses from sylvatic environments [24]. Interestingly, we did not detect marsupials as food sources but the eight *R. rattus*, an exotic species whose occurrence is restricted to the artificial environment, identified as a food source for *T. sordida* specimens were infected by TcI. Furthermore, one insect that fed on *T. apereoides* was infected with TcI, while the other two bugs that fed on this rodent were infected with TcII and TcV. Both DTUs were identified much more rarely, with TcII being present in a higher proportion compared to TcV [64]. These DTUs are more often associated with domestic cycles in South America [64,65], though their natural hosts remain poorly defined. In Bolivia, TcII and TcV are the predominant genotypes detected in Rodentia [64]. Regarding the TcIII, this DTU was reported in *T. sordida* from peridomestic environments in Bahia, Brazil, being consistent with our findings [66]. It is important to highlight that TcIII may be underreported in both domestic and wild cycles, because some typing methodologies fail to distinguish between TcIII and TcIV [67].

We observed a significant difference in parasitic load across developmental stages, with females exhibiting a higher *T. cruzi* load compared to N3, N4, N5 instars, and males. Females of *T. melanica* from Espinosa, MG, also exhibited the highest parasitic load when compared to nymphs [31]. The ovarian development and egg production in triatomines are highly dependent on blood meals [68]. *T. sordida* inhabits less stable ecotopes and utilizes a wide range of food sources and allocates more energy to reproduction than maintenance [16,69]. This increased energy investment in reproduction likely elevates the probability of using several food sources, which may contribute to the higher parasitic load in females. However, to prove this correlation, it is necessary to design and develop experimental infection studies under laboratory conditions.

It is important to highlight that *T. sordida* defecates during blood meals, demonstrating its strong vector competence for Chagas disease [70]. Although the parasitic load detected in *T. sordida* was not the highest compared to other triatomine species investigated so far, our findings indicate that this species deserves greater attention due to its high *T. cruzi* infection rate. Furthermore, the diversity of blood meal sources and *T. cruzi* DTUs underscores the intense circulation of different hosts in peridomestic ecotopes, facilitating the overlap between natural and artificial environments. In this regard, the findings of this study have significant implications for vector control, indicating the need to maintain control and surveillance measures, considering the local dynamics of vectors and hosts.

## 5. Conclusions

The high rates of natural infection by *T. cruzi* observed in *T. sordida*, combined with frequent reports of its prevalence in peridomestic environments, underscore the epidemiological importance of this vector. Despite the parasitic load of *T. sordida* being lower than that of other species previously quantified, this finding emphasizes the underdetection of positive specimens by optical microscope, the commonly employed technique in routine entomological surveillance. This study also confirms the predominance of chickens as food sources and their role in the epidemiological scenario of *T. cruzi* transmission. Although chickens are considered refractory to the parasite, they serve as food sources, sustaining previously infected insects. Wild and synanthropic rodents, along with other mammals identified as food sources, frequently invade peridomestic structures, especially chicken coops, attracted by chicken feed and droppings. Consequently, these animals act as sources of *T. cruzi* infection for the *T. sordida* population. Furthermore, the great diversity of *T. cruzi* DTUs detected in triatomine specimens captured in artificial ecotopes is likely due to the intense circulation of different hosts in wild and domestic habitats, as well as triatomine migration, leading to overlapping DTU transmission cycles.

## Figures and Tables

**Table 1:** Number of nymphs (N1–N5) and adults (F, M) of *Triatoma sordida* collected at different locations in the rural region of Espinosa, MG, Brazil (2018–2019), and their infection by *Trypanosoma cruzi* and the respective infection rates (%).

	N1			N2			N3			N4			N5			F			M		
Ecotope	C	E	I	C	E	I	C	E	I	C	E	I	C	E	I	C	E	I	C	E	I
Indoor	18	1	0	12	4	0	20	17	1 (5.9)	17	15	0	12	12	1 (8.3)	3	2	0	2	2	0
Chicken coop	58	1	0	146	31	2 (6.4)	123	111	15 (13.5)	160	153	13 (8.5)	468	442	87 (19.7)	238	190	25 (13.1)	292	225	26 (11.5)
Pigsty	1	1	1 (100)	10	4	1 (25)	8	5	3 (60)	12	10	1 (10)	60	60	14 (23.3)	11	11	1 (9)	12	12	3 (25)
Wood pile	5	2	0	10	2	1 (50)	15	10	1 (10)	24	24	3 (12.5)	27	26	5 (19.2)	5	4	0	2	2	0
Tile pile	1	0	0	3	0	0	5	3	0	4	4	1 (25)	22	21	2 (9.5)	8	7	0	18	18	0
Brick pile	0	0	0	0	0	0	1	1	0	1	1	1 (100)	2	2	0	2	2	1 (50)	3	3	1 (33.3)
Piles of tile/brick	0	0	0	5	0	0	0	0	0	4	3	0	4	4	0	1	1	0	0	0	0
Corral	0	0	0	0	0	0	1	1	0	0	0	0	2	2	0	1	1	0	2	2	0
Total	83	5	1 (20)	186	41	4 (9.7)	173	148	20 (13.5)	222	210	19 (9)	597	569	109 (19.1)	269	218	27 (12.4)	331	264	30 (13.4)

C = number of captured insects; E = number of examined insects by optical microscope and/or PCR; I, (%) *=* number and percentages of triatomines infected by *T. cruzi*; N1–N5 = first to fifth nymphal instar; F = females; M = males.

**Table 2:** Number of *Triatoma sordida* specimens collected at different ecotopes in the rural region of Espinosa, MG, Brazil (2018–2019), and their infection by *Trypanosoma cruzi* and the respective infection rates (%).

Ecotopes	E	I
Indoor	53	2 (3.8)
Chicken coop	1153	168 (14.6)
Pigsty	103	24 (23.3)
Wood pile	70	10 (14.3)
Tile pile	53	3 (5.7)
Brick pile	9	3 (33.3)
Piles of tile/brick	8	0
Corral	6	0
Total	1.455	210 (14.4)

E = number of examined insects by MO and/or PCR; I (%) = number and percentages of triatomines infected by *T. cruzi*.

**Table 3:** Distribution of *T. cruzi* DTUs and mixed infections in *T. sordida* specimens according to capture ecotopes in the studied areas of the rural region of Espinosa, MG, Brazil.

	TcI	TcII	TcIII	TcV	TcI + TcII	TcI + TcIII	TcI + TcV
Indoor	1	0	0	0	0	0	0
Chicken coop	98	3	3	6	2	1	1
Pigsty	7	9	1	1	0	0	0
Wood pile	6	0	0	0	0	0	0
Tile pile	0	0	1	0	0	0	0
Brick pile	2	0	0	0	0	1	0
Total	114	12	5	7	2	2	1

**Table 4:** Number of infections of *T. sordida* with different DTUs after feeding on different hosts.

	TcI	TcII	TcIII	TcV	TcI + TcII	TcI + TcIII
*Gallus gallus*	48	10	5	6	1	2
*Thrichomys apereoides*	1	1	0	1	0	0
*Capra hircus*	1	0	0	0	0	0
*Felis catus*	1	0	0	0	0	0
*Rattus rattus*	8	0	0	0	0	0
*Sus scrofa*	3	0	0	0	0	0
*Canis lupus familiaris*	2	0	0	0	0	0
Total	64	11	5	7	1	2

## Data Availability

Data are included within the article. Sequence data have been deposited in GenBank.
